# Case Report: Behçet’s syndrome and venous thrombosis in children: a case study and review of the literature

**DOI:** 10.3389/fimmu.2025.1664004

**Published:** 2025-09-29

**Authors:** Qinan Yin, Yuan Bian, Fang Niu, Mao Zhang, Siyu Chen

**Affiliations:** ^1^ Department of Pharmacy, Personalized Drug Research and Therapy Key Laboratory of Sichuan Province, Sichuan Provincial People’s Hospital, School of Medicine, University of Electronic Science and Technology of China, Chengdu, China; ^2^ Department of Vascular Surgery, Sichuan Academy of Medical Sciences & Sichuan Provincial People’s Hospital, School of Medicine, University of Electronic Science and Technology of China, Chengdu, China

**Keywords:** Behçet’s syndrome, child, venous thrombus embolism, anticoagulation, treatment

## Abstract

Venous thromboembolism (VTE) is an exceedingly rare occurrence in pediatric patients with Behçet’s syndrome (BS). Currently, the clinical anticoagulation treatment regimen for this condition in the pediatric population remains inadequately defined. Here, we reported the treatment process of a child with Behçet’s disease complicated by VTE and reviewed the relevant literature to study comprehensive diagnosis and treatment strategies, as well as the latest progress for this specific type of disease. A 15-year-old boy who presented BS with pulmonary embolism, pulmonary aortic aneurysm, and recurrent deep vein thrombosis was described. Following a comprehensive strategy of systematic immunotherapy and anticoagulation treatment, the patient’s condition has shown significant improvement, with marked relief from pulmonary embolism and lower extremity venous thrombosis. In addition to the systematic immunotherapy, anticoagulant therapy plays a significant role in the diagnosis and treatment of these patients. The literature review shows that 16 of the 17 patients underwent heparin anticoagulant therapy, while 6 were additionally treated with vitamin K antagonists (VKs). Our case study also indicated that vitamin K antagonists can be applied in this population. Moreover, it is imperative to conduct extensive long-term follow-up studies to better evaluate the outcomes of anticoagulation therapy as these patients transition into adulthood.

## Introduction

BS is a chronic, recurrent autoimmune and inflammatory disorder characterized by a range of clinical manifestations, including oral and genital ulcers, skin lesions, and potential involvement of the eyes, blood vessels, joints, gastrointestinal tract, reproductive system, and other organs (1). This condition predominantly affects young adults aged 20 to 40; however, it is also diagnosed in pediatric populations, with 4% to 26% of cases occurring in individuals under 16 years of age, a condition referred to as pediatric Behçet’s disease (2, 3). VTE represents the most prevalent form of vascular involvement in Behçet’s disease. The incidence of vascular involvement in pediatric patients with Behçet’s disease ranges from 1.8% to 21.0% (4). Thromboembolic events in these patients may present in atypical anatomical locations and can extend to the pulmonary artery and multiple veins (5). Given the rarity of pediatric Behçet’s disease, thrombotic complications associated with the syndrome are also uncommon (6). Currently, diagnostic and therapeutic approaches are primarily based on guidelines developed for adults, resulting in a lack of pediatric-specific pharmacological treatment experience, particularly concerning anticoagulation therapy for BS and associated thrombosis. An examination of the current literature reveals that it predominantly consists of case reports and small case series. The diagnosis, treatment, particularly the anticoagulation management, and outcomes for pediatric patients with BS complicated by VTE are insufficiently documented. This article presents a rare case of pediatric Behçet’s syndrome with venous thromboembolism and undertakes an literature review to investigate and elucidate the prevailing anticoagulation strategies for children with BS and VTE.

## Methods

A child with BS and multiple venous thromboses was presented. A comprehensive literature search was conducted in PubMed from its inception to 30 April 2025. The keywords used in the search were “Behcet’s syndrome,” “Behcet’s disease,” “child,” “children,” “pediatric,” “venous thromboembolism,” “venous thrombus,” “venous thrombosis,” “deep vein thrombosis,” and “pulmonary embolism,” associated with AND/OR. References for the retrieved studies were reviewed to identify additional reports. Only pediatric cases (aged 16 years or younger) that provided sufficient detail for individual analysis were included.

## Results

### Case report

A 15-year-old male patient presented with PE and a pulmonary aortic aneurysm secondary to hemoptysis, as well as recurrent DVT of the left lower extremity, diagnosed one week later. Upon inquiry into his medical history, the patient reported experiencing recurrent oral ulcers for three years prior to admission, occurring more than three times annually, and occasional erythema in the lower limbs, which resolved spontaneously. He denied any abnormal vision, headaches, arthralgia, nausea, vomiting, or other symptoms of discomfort. Physical examination upon admission revealed multiple scattered oral mucosal ulcers, each less than 1 cm in diameter, and superficial thrombophlebitis in the left lower limb, accompanied by varicose veins. Additionally, there was erythema surrounding the superficial venous skin, mild edema of the left lower limb, a diminished dorsalis pedis pulse, and a slightly elevated skin temperature compared to the right lower limb. No significant abnormalities were observed in the upper limbs, and there were no genital ulcers. The patient reported no relevant family medical history. Laboratory tests showed elevated C-reactive protein (31.56 mg/L), D-dimer (1.61 μg/mL), and platelet counts (581 × 10^9^/L). During hospitalization, hematologic examinations, including a bone marrow smear and abnormal cell screening, were performed, and hematologic tumors were excluded. Further examination revealed an erythrocyte sedimentation rate of 24 mm/h, increased coagulation factor VIII activity (153.4%), normal plasma protein C and S activity, antinuclear antibody, anticardiolipin antibody, and ferritin levels. Chest enhanced CT demonstrated pulmonary artery malformation with thrombosis in the upper dorsal segment of the left lower lobe, aneurysmal dilatation of the main pulmonary artery in the right lower lobe with embolus formation, and emboli in the branches of the pulmonary arteries in both lower lobes. Echocardiographic evaluation revealed the presence of mild tricuspid valve insufficiency. Following a consultation with a rheumatologist and based on the 2015 classification criteria for pediatric Behçet’s disease (PEDBD) (7, 8), the patient, who exhibited recurrent oral ulcers, venous thrombosis, pulmonary aneurysm, and superficial thrombophlebitis of the lower extremities, was diagnosed with BS.

**Figure 1:**
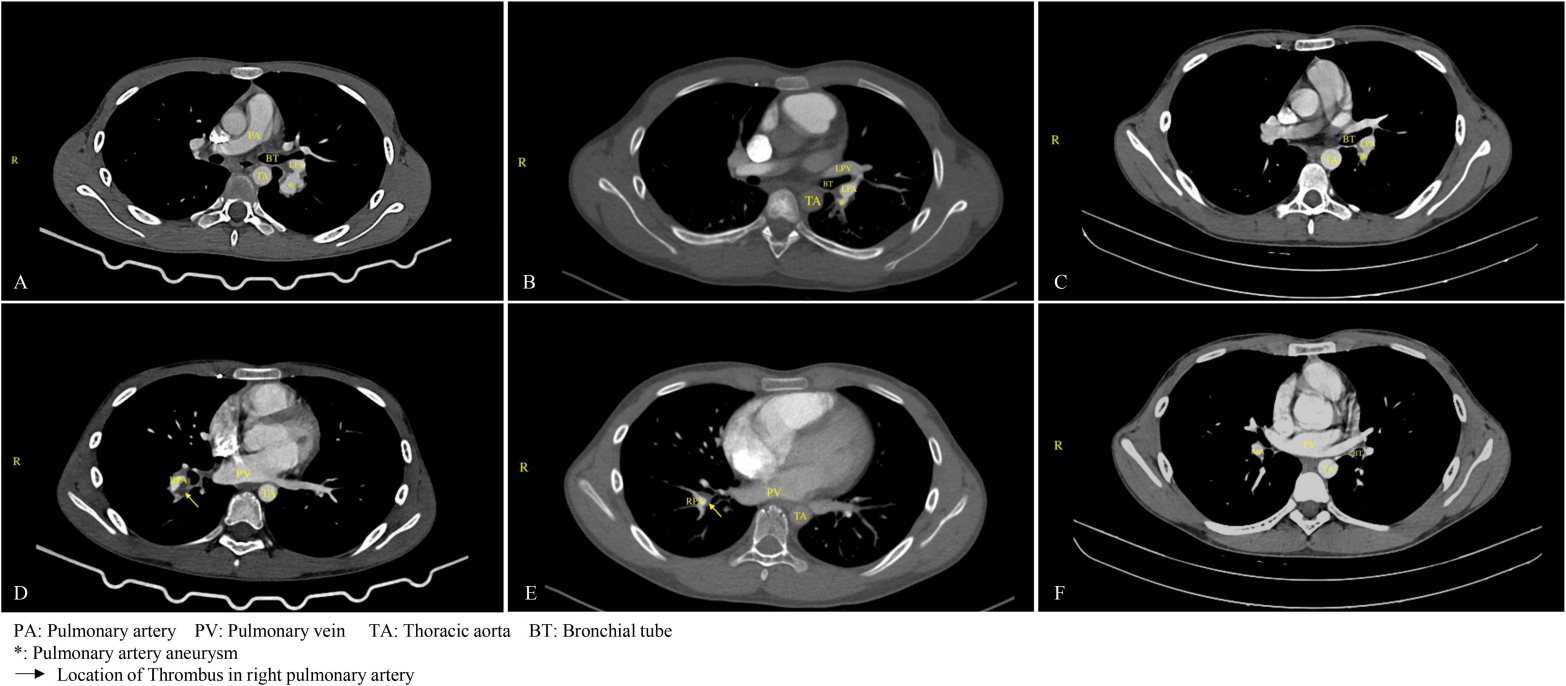
Changes in pulmonary aneurysm and thrombus before and after treatment. **(A)** On March 18, 2024, the patient was diagnosed with a pulmonary aneurysm. **(B)** A follow-up examination on August 1, 2024, indicated no significant change in the patient's pulmonary aneurysm compared to the previous assessment. **(C)** By August 4, 2025, a follow-up examination of the child revealed a significant reduction in the aneurysm. **(D)** On March 18, 2024, thrombosis was identified in the upper posterior segment of the left lower lobe, along with aneurysmal dilation of the pulmonary artery in the right lower lobe accompanied by thrombus formation, and thrombus formation in the branches of the pulmonary arteries in both lower lobes. **(E)** After four months of anticoagulation treatment, as of August 1, 2024, the thrombus in the main pulmonary artery had resolved, although embolization persisted in a small branch of the pulmonary artery in both lower lobes. **(F)** By August 4, 2025, a follow-up examination of the child confirmed the resolution of the pulmonary thrombus.

Hemoptysis was treated with bronchial embolization. Medical treatment was initiated with subcutaneous enoxaparin, high-dose oral prednisone (40mg qd), and diosmin to improve the leg heaviness. During treatment, color Doppler ultrasound showed reduced thrombosis in the left external iliac and common femoral veins with partial recanalization. Warfarin monotherapy continued after the INR reached the target range. At discharge, the rash and oral ulcers had resolved. After that, low-dose prednisone and long-term warfarin anticoagulation were administered. Two months following discharge, the patient consulted with a rheumatologist, who strongly recommended the administration of adalimumab, an anti-tumor necrosis factor (TNF) agent. However, this recommendation, along with the suggestion to use cyclophosphamide, was declined by the patient’s guardian. After extensive discussions with the patient’s family, azathioprine was selected as an alternative for immunotherapy and has been administered continuously to date. A comprehensive overview of the medical regimen is detailed in **Table 1**. Throughout the six-month follow-up period, the child’s daily academic and extracurricular activities remained unaffected. A computed tomography angiography (CTA) of the lungs conducted four months post-discharge revealed the resolution of the thrombus in the main pulmonary artery, with only minor embolisms persisting in a few small branches. A subsequent CT scan performed one year later indicated the complete resolution of the pulmonary embolism, with no significant alterations observed in the venous thrombosis of the left lower extremity (**Figure 1**).

**Table 1:** Patient medication list.

Drug name	Rationale	Dosage & Administration	Duration
Enoxaparin Sodium Injection	Treatment of VTE	4000 IU Q12H Subcutaneous	12 days
Diosmin Tablets (Citrus Flavonoids)	Improve lower limb swelling and discomfort	500 mg BID Oral	28days
Omeprazole Enteric-Coated Capsules	Prevent steroid and anticoagulant-induced stress ulcer	10 mg QD Oral	18days
Prednisone Acetate Tablets	Immunosuppressive therapy	40 mg QD Oral	4 months
Warfarin Sodium Tablets	Long-term treatment of VTE	3.75 mg QD Oral	1 year till now
Azathioprine Tablets	Immunosuppressive therapy	100 mg QD Oral	8 months till now
Prednisone Acetate Tablets	Immunosuppressive therapy	10 mg QD Oral	8 months till now

### Literature review

In addition to our case, 18 reports of VTE in children with BS were identified, and one case involving death was excluded. The remaining 17 patients are presented in **Table 2**. The median age of the patients was 12 years (range, 4–15 years), and 82.3% (n=14) were male. Among the 17 patients, only two were diagnosed with BS first and thrombosis later. Nine patients (52.9%) had DVT, two (11.7%) had PE, two (11.7%) had inferior vena cava thrombosis (IVCT), and four (23.5%) had both DVT and PE. Only 1 patient with a pulmonary aortic aneurysm and PE also had DVT. Six patients had cerebral venous sinus thrombosis (CVST) involvement; in some cases, CVST was the first manifestation of thrombosis. In addition, hepatic vein thrombosis and right atrial thrombosis occurred in two patients. In these patients, the most common manifestation was oral ulcer (11 patients, 64.7%), followed by arthritis or joint pain (8 patients, 47%), and erythema nodosum (6 patients, 35.3%). One patient had a complete loss of vision in the right eye. Colchicine was administered to 82.3% (n=14) of patients, and corticosteroids were administered in the same proportion. Cyclophosphamide was used in 47% (n=8) of the patients, infliximab in 23.5% (n=4), methotrexate in 5.8% (n=1), and mycophenolate mofetil in 5.8% (n=1). Of the 17 patients, 16 underwent definitive anticoagulation therapy, and all received heparin anticoagulants. Additionally, 6 patients were treated with vitamin K antagonists. Detailed records of anticoagulation duration were available for 8 patients, with treatment durations ranging from 3 months to 2 years. The follow-up period for these patients ranged from five months to six years. Two patients experienced thrombus resolution; however, their follow-up periods were relatively short (five and six months, respectively). Recurrent thrombosis occurred in three patients.

**Table 2:** Clinical characteristics of reported cases of VTE associated with paediatric Behçet’s disease.

Age	Sex (M/F)	Known BS	Type of VTE	Additional vascular involvement	Anticoagulation and duration		Thrombotic outcome	Other treatment	Other involvement	Followup	Ref
15	M	No	DVT, PE	None	Enoxaparin, warfarin	Up to now (>6 months)	recanalize	high-dose oral prednisone, diosmin , azathioprine	oral ulcers, erythema nodosum	6 months	Our case
12	M	No	DVT	CVST	LMWH, warfarin	NA	recanalize	steroids, colchicine, azathioprine	oral ulcers, skin rash	6 months	(9)
11	M	No	IVCT	CVST	LMWH, VKA	2 years	NA	colchicine	erythema nodosum, folliculitis	64 months	(10)
8	M	No	DVT	None	Enoxaparin, heparin, warfarin	6 months	recurrence (developed recurrent bilateral lower extremity thrombi)	prednisone, methotrexate, infliximab	erythema nodosum,	5 years	(11)
12	M	No	DVT, PE	CVST, pulmonary artery aneurysm	LMWH, warfarin	1.5 years	NA	prednisolone, cyclophosphamide, mycophenolate mofetil, aspirin	mucosal ulcers (tongue), both eyes were at rophied, leaving irreversible visual loss in the right eye	2 years	(12)
4	F	No	DVT	None	LMWH, heparin,	5 months	recanalize	colchicine	oral ulcers, arthritis	5 months	(13)
15	F	No	IVCT	CVST, hepatic vein thrombosis	NA	NA	recanalize	prednisolone, infliximab, cyclophosphamide	papilledema	6 months	(14)
5	M	No	DVT	RVT	LMWH	6 months	NA	colchicine, corticosteroids, azathioprine, or cyclophosphamide	arthralgia	16 months	(6)
15	M	No	DVT, PE	None	LMWH	6 months	NA	colchicine, corticosteroids, azathioprine, or cyclophosphamide	oral ulcers	19 months	(6)
14	M	Yes	DVT	None	LMWH	3 months	NA	colchicine, corticosteroids, azathioprine, or cyclophosphamide	arthralgia	26 months	(6)
8	M	No	DVT	None	heparin, warfarin	NA	NA	colchicine, prednisone, azathioprine	arthralgia, abdominal pain, oral aphthous lesions, and uveitis	NA	(15)
14	M	No	DVT	None	heparin, dicoumarin	3 months	NA	colchicine	oral ulcers, gingivitis	NA	(16)
7	M	Yes	PE	CVST, right Atrial thrombus	Enoxaparin	NA	recurrence	pulse methylprednisolone, cyclophosphamide, colchicine, infliximab	oral ulcers	2 years	(17)
13	M	NA	DVT, PE	None	Enoxaparin	NA	recanalize	colchicine, azathiopurine, prednisolone	oral ulcers, genital ulcers, acne, arthralgia	5 years	(17)
12	F	NA	DVT	None	Enoxaparin	NA	recanalize	colchicine, azathiopurine, prednisolone	oral ulcers, genital ulcer, acne, arthralgia	6 years	(17)
13	M	NA	DVT	None	Enoxaparin	NA	recanalize	colchicine, prednisolone	oral ulcers, acne, erythema nodosum, arthralgia	5 years	(17)
14	M	NA	DVT, PE	None	Enoxaparin	NA	recanalize	colchicine, azathiopurine, prednisolone	oral ulcers, acne, erythema nodosum, arthralgia	4 years	(17)
11	M	NA	PE	CVST	Enoxaparin	NA	recurrence	colchicine, pulse methylprednisolone, prednisolone, cyclophosphamide, infliximab	oral ulcers, erythema nodosum, acne, uveitis, epididymitis, arthritis, decreased visual acuity	2 years	(17)

NA, not available; DVT, deep vein thrombosis; PE, pulmonary embolism; CVST, cerebral sinus vein thrombosis; IVCT, Inferior vena cava thrombosis; LWMH, low-molecular weight heparin; VKA, vitamin K antagonist; Known BS, Patients were diagnosed with BS before developing thrombosis.

## Discussion

BS is a rare multisystem inflammatory disorder characterized by diverse clinical manifestations, recurrent episodes, and chronic progression, with a tendency toward more severe progression in males and young adults (18–20). Male patients are more likely to develop vascular complications (21), particularly affecting cerebral and lower extremity vessels (22). Pediatric BS demonstrates distinct phenotypic clusters compared to adult-onset disease, and a Chinese cohort study identified three subtypes in children (23). Vascular and neurological manifestations are generally rare in children (24). A prospective cohort reported venous thrombosis in 9.6% of pediatric BS cases, with arterial thrombosis or aneurysms occurring in only 1.8% (25). However, a Turkish study noted a higher incidence of mixed vascular disease, which can lead to severe complications, including death (26). According to the 2024 EUROFEVER registry, venous thromboses accounted for 70% of events in pediatric BS, with cerebral venous sinus thrombosis (44%) and lower extremity DVT (23%) being the most common (27). But it’s uncommon to diagnose a pediatric BS patient accompanied by PE, a pulmonary aortic aneurysm, and DVT at the same time. Among 17 documented pediatric cases, only one other patient, aged 12, demonstrated similar vascular complications, which also became symptom-free by anticoagulation therapy (12).

Since thrombosis in BS is primarily driven by vascular inflammation, immunosuppressive therapy leads to resolution or regression of aneurysms or thrombosis in approximately 70% of patients (28), making it the cornerstone of acute venous thrombosis management (29). The role of anticoagulation remains controversial (30). In this review, all but one patient received anticoagulants, and only three experienced recurrent thrombosis. A meta-analysis cited in current guidelines found no significant benefit from adding anticoagulation to immunosuppression in preventing relapse (31). Nonetheless, French guidelines recommend full anticoagulation for DVT in both adult and pediatric BS (32). A retrospective study showed that combined anticoagulant and immunosuppressive therapy significantly reduced thrombosis recurrence compared to immunosuppression alone (33), a finding supported in another study focusing on PE (34). Our case also used anticoagulation therapy combined with immunosuppressive therapy, which achieved a great effect. In all reported cases, patients received heparin or warfarin; no direct oral anticoagulants (DOACs) were used. However, experience in adults suggests that DOACs may reduce recurrent thrombosis risk and synergize with disease-modifying antirheumatic drugs (DMARDs) (35). Although dabigatran and rivaroxaban are approved for some pediatric indications, their use in BS remains undocumented and may represent a future therapeutic avenue. In our case, based on previous guidelines and literature reviews, we also chose warfarin as long-term anticoagulant therapy.

The primary treatment goals in BS are to control inflammation rapidly, prevent relapses and irreversible organ damage, and slow disease progression. Management must be individualized according to age, gender, and organ involvement type and severity (36). First-line agents include corticosteroids, immunomodulators, and immunosuppressants (37). Since thrombosis in BS is primarily driven by vascular inflammation, immunosuppressive therapy leads to resolution or regression of aneurysms or thrombosis in approximately 70% of patients (28), making it the cornerstone of acute venous thrombosis management (29). The 2018 EULAR recommendations advise using azathioprine, cyclophosphamide, or cyclosporine for vascular involvement, including acute DVT, with anti-TNF agents reserved for refractory cases (38). Although glucocorticoids and cyclophosphamide are first-line, anti-TNF agents have shown efficacy in refractory cases (39). In a study examining the application of biological agents in pediatric patients with Behçet’s disease, anti-TNF agents emerged as the most frequently utilized biologic agents. The primary indication for employing these biologic agents was ocular involvement, followed by active multisystem disease. While both agents have demonstrated efficacy in treating Behçet’s syndrome in children and possess an acceptable safety profile, there remains a need for controlled studies to further investigate the appropriate indications for biotherapy in this population (40). In the present case, azathioprine was selected after shared decision-making with the family, considering the high cost and lack of reimbursement for anti-TNF agents in China for pediatric BS. Combined with warfarin, this regimen resulted in regression of pulmonary thrombosis and favorable outcomes during over one year of follow-up. Notably, nearly one-third of pediatric BS patients may develop new major organ involvement or relapse in adulthood (25), underscoring the need for long-term follow-up. Another barrier to anti-TNF use is that neither infliximab nor adalimumab is approved in China for pediatric BS, further limiting treatment options. Currently, no specific guidelines exist for managing vascular BS in children, and large-scale studies are lacking. The potential for recurrent thrombosis remains a concern.

Although the efficacy of enoxaparin and warfarin in preventing thrombotic events remains uncertain, this case is particularly noteworthy and valuable due to the ongoing follow-up and significant improvement in the patient’s thrombotic condition. This case emphasizes the importance of screening for rheumatologic diseases—including Behçet’s, antiphospholipid syndrome, and systemic lupus erythematosus—in children with unprovoked DVT, even when mild thrombophilia is detected (11, 41). Screening should include a detailed history, physical examination for rheumatic features, and inflammatory and immune markers (e.g., ESR, CRP, ANA) (11). In our case, only anti-cardiolipin antibody (aCL) was tested; lupus anticoagulant (LA) was not performed—a study limitation. Simultaneous testing for both LA and aCL is recommended for more comprehensive evaluation in future cases.

## Conclusions

The occurrence of BS complicated by DVT and PE is uncommon in pediatric populations, and the combination of DVT, PE, and pulmonary aneurysm is even rarer. The use of anticoagulants in such cases remains controversial and complicates treatment strategies. This case may serve as a reference for clinical management of similar pediatric presentations. Future multicenter controlled trials are needed to provide further insights and evaluations.

## Data Availability

The original contributions presented in the study are included in the article/supplementary material. Further inquiries can be directed to the corresponding author.
